# The symmetry of the left and right tibial plateau: a comparison of 200 tibial plateaus

**DOI:** 10.1007/s00068-022-02043-5

**Published:** 2022-07-13

**Authors:** Nynke van der Gaast, Hans Dunning, Jellina M. Huitema, Andrew Waters, Ruurd L. Jaarsma, Job N. Doornberg, Michael J. R. Edwards, Sebastiaan A. W. van de Groes, Erik Hermans

**Affiliations:** 1 Department of Trauma Surgery, Radboud University Medical Center, Geert Groteplein Zuid, 6525 GA Nijmegen, the Netherlands; 2 Orthopaedic Research Laboratory, Radboud Institute for Health Sciences, Radboud University Medical Center, Nijmegen, The Netherlands; 3 Department of Orthopaedic Surgery, Radboud University Medical Center, Nijmegen, the Netherlands; 4 Department of Orthopaedic and Trauma Surgery, Flinders Medical Centre, Adelaide, Australia; 5 Department of Orthopaedic Surgery, University Medical Center Groningen, Groningen, the Netherlands

**Keywords:** Tibial plateau, Tibial plateau fractures, Symmetry, 3D virtual planning, Surgical planning

## Abstract

**Purpose:**

This study aims to investigate the symmetry of the left and right tibial plateau in young healthy individuals to determine whether left–right mirroring can be reliably used to optimize preoperative 3D virtual planning for patients with tibial plateau fractures.

**Methods:**

One hundred healthy subjects, without previous knee surgery, severe knee trauma, or signs of osteoarthritis were included for a previous dynamic imaging study of the knee. The subjects underwent a CT scan, scanning the left and right knee with a slice thickness of 0.8 mm. 3D surface models of the femur, patella, and tibia were created using a convolutional neural network. The 3D models of the left and right tibias were exported to MATLAB © and the tibias were mirrored. The mirrored tibias were superimposed on the contralateral tibia using a coherent point drift surface matching algorithm. Correspondence points on both surfaces were established, the mean root squared distance was calculated and visualized in a boxplot and heatmaps.

**Results:**

The overall mean difference between correspondence points on the left and right tibial plateau is 0.6276 ± 0.0343 mm. The greatest differences between correspondence points were seen around two specific surfaces on the outside of the tibial plateau; where the distal tibia was cut 15 mm below the tibial plateau and around the tibiofibular joint.

**Conclusions:**

The differences between the left and right tibial plateau are small and therefore, we can be confident that the mirrored contralateral, unfractured, tibial plateau can be used as a template for 3D virtual preoperative planning for young patients without previous damage to the knee.

## Introduction

The tibial plateau is one of the crucial weight-bearing areas of the body. Fractures of the tibial plateau are intra-articular and therefore often technically challenging to treat. A bimodal distribution is seen in age; high-energetic trauma for younger patients in contrast to relatively low-energetic traumas in older patients with osteoporosis [1]. Patients with tibial plateau fractures are highly susceptible to complications including knee stiffness, posttraumatic osteoarthritis, and non- or mal-union [2]. Anatomic reconstruction of the articular surface is key to prevention of these complications. Recognition and understanding of the fracture and its fracture lines are crucial for determining the optimal surgical approach for fracture reduction [3]. Preoperative planning could be important for the patients’ prognosis, and the choice of surgical technique has proven to be of impact on the functional recovery of the knee according to recent studies [3–5].

Currently, radiographs and two- and three-dimensional (3D) computed tomography are used for surgical planning [6–9]. Since these images are static and virtual reduction is not possible, it can be difficult for surgeons to create an optimal strategy for surgical reduction. Consequently, surgeons are continuously looking for improvements in preoperative planning when treating complex fractures. Three-dimensional (3D) virtual planning is a relatively new tool that might improve the insight into fracture characteristics and thereby improve fracture reduction and decrease complications, blood loss, and operating time [4, 10, 11]. 3D virtual planning can be provided by expert programs, such as Sectra Medical Systems AB^©^ (Linköping, Sweden) and Materialise^©^ (Leuven, Belgium). These programs are gaining popularity and the additional value of these programs is currently being investigated.

For surgical planning, the contralateral, unfractured tibial plateau, is already used as a template for optimal reduction of the fractured tibial plateau [3, 12]. Several studies have been performed on assessing limb symmetry using different methods [13–16]. In a study by Quintens et al. [15], statistical shape modeling was used to gain insight into anatomical variations of the tibia using a principal component analysis based on five parameters of the tibia. Small differences in shape variation were found between the left and right tibial plateau. Whilst this demonstrates that there is a difference in shape variation within a population, it is less indicative of the left–right difference within one patient. Similarly, a study by Jang et al. [16] compared 3D morphometric measurements on ten fresh frozen cadavers and found small within-subject differences of 1.1 ± 0.6 mm between the left and right proximal tibia of one subject. Although both previous named studies suggest a small difference between the left and right tibia, they can only draw a limited conclusion because of indirect left–right comparison, high age of participants, and small sample sizes. Therefore, we aim to investigate the symmetry of the left and right tibial plateau in young healthy individuals to determine whether left–right mirroring can be used to optimize preoperative 3D virtual planning for patients with tibial plateau fractures.

## Methods

**Fig. 1:**
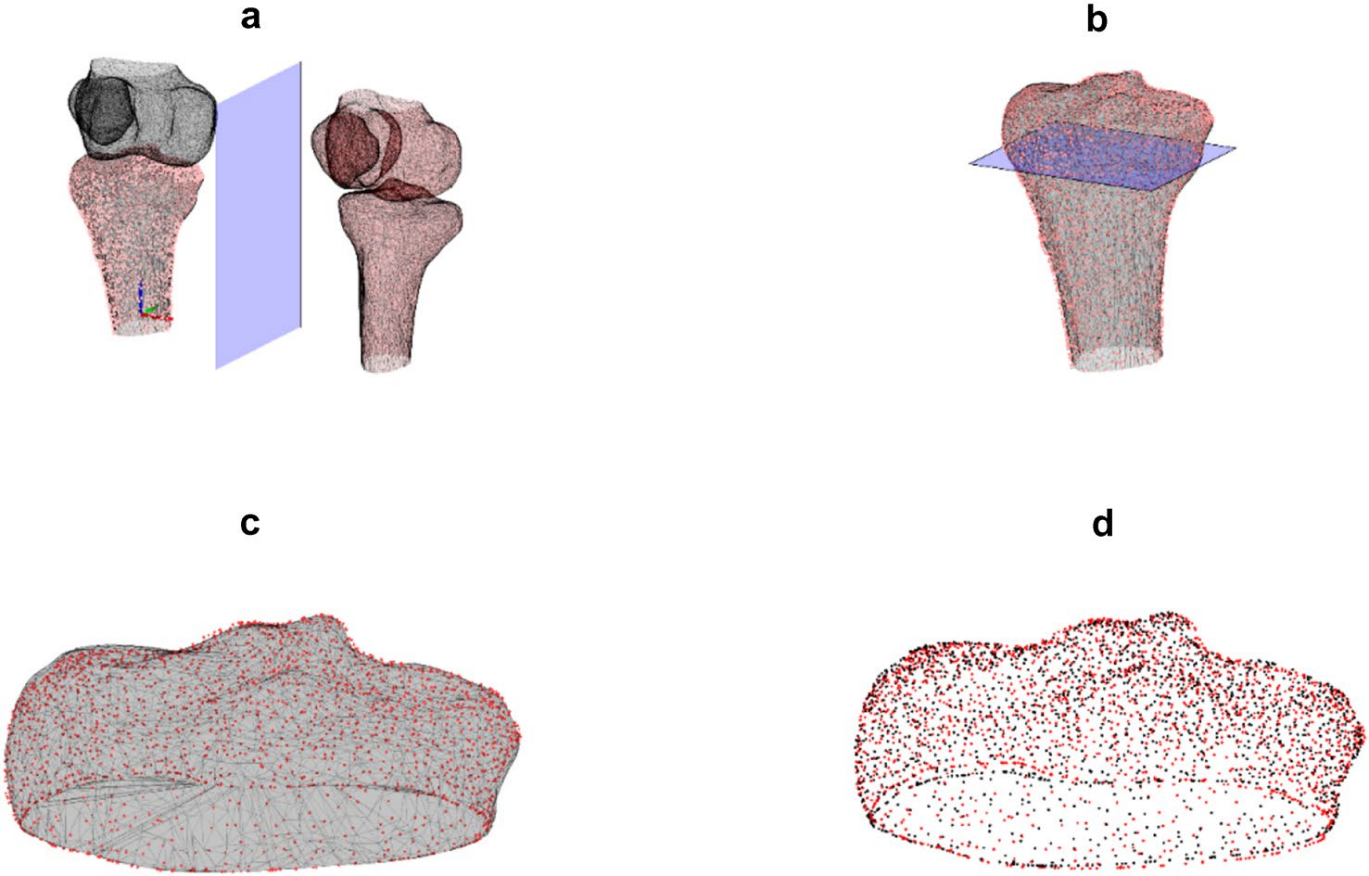
Overview of methods. **a** Left tibia (red) is mirrored along the sagittal plane (blue). The surface of the mirrored left tibia is superimposed with the surface of the right tibia (black). **b** Superimposed, mirrored left tibia and right tibia are cut 15 mm below the tibial plateau in an axial plane (blue). **c** Resulting proximal parts of both tibias are again superimposed to prevent malposition due to distal surface points. **d** Correspondence points are established (red and black) and the Euclidean distance between these points is calculated

Data for this study was collected for a previous study on dynamic, four-dimensional (4D), imaging of the knee, which was approved by our local ethics committee (Ethics approval number: NL 72784091). The secondary use of this data was approved by all subjects in a written informed consent file. The procedures used in this study adhere to the tenets of the Declaration of Helsinki. One hundred healthy subjects, without previous knee surgery, severe knee trauma, or signs of osteoarthritis, were included. In the context of the ongoing imaging study, healthy individuals underwent a CT scan (Canon Aquilion One), scanning both knees with a slice thickness of 0.8 mm. The images had voxel sizes of 0.782 × 0.78 × 20.8 mm. For this study, 3D surface models of the femur, patella, and tibia were created using a convolutional neural network [17]. The 3D models of the left and right tibias were exported to MATLAB^©^(The MathWorks Inc, Natick, Massachusetts, United States). The left tibias for each participant were mirrored in the sagittal plane. The mirrored left tibias were superimposed on the contralateral right tibia using a computer-based Coherent Point Drift surface matching algorithm [18]. The target and superimposed surface models were cut 15 mm below the tibial plateau. The resulting surfaces were again superimposed to ensure alignment of the proximal tibia and to avoid point drift due to points outside our region of interest. Correspondence points were identified on both surfaces. The root mean squared distance between correspondence points on both surfaces was calculated in millimeters and visualized in heatmaps (Fig. 1).

## Results

**Fig. 2:**
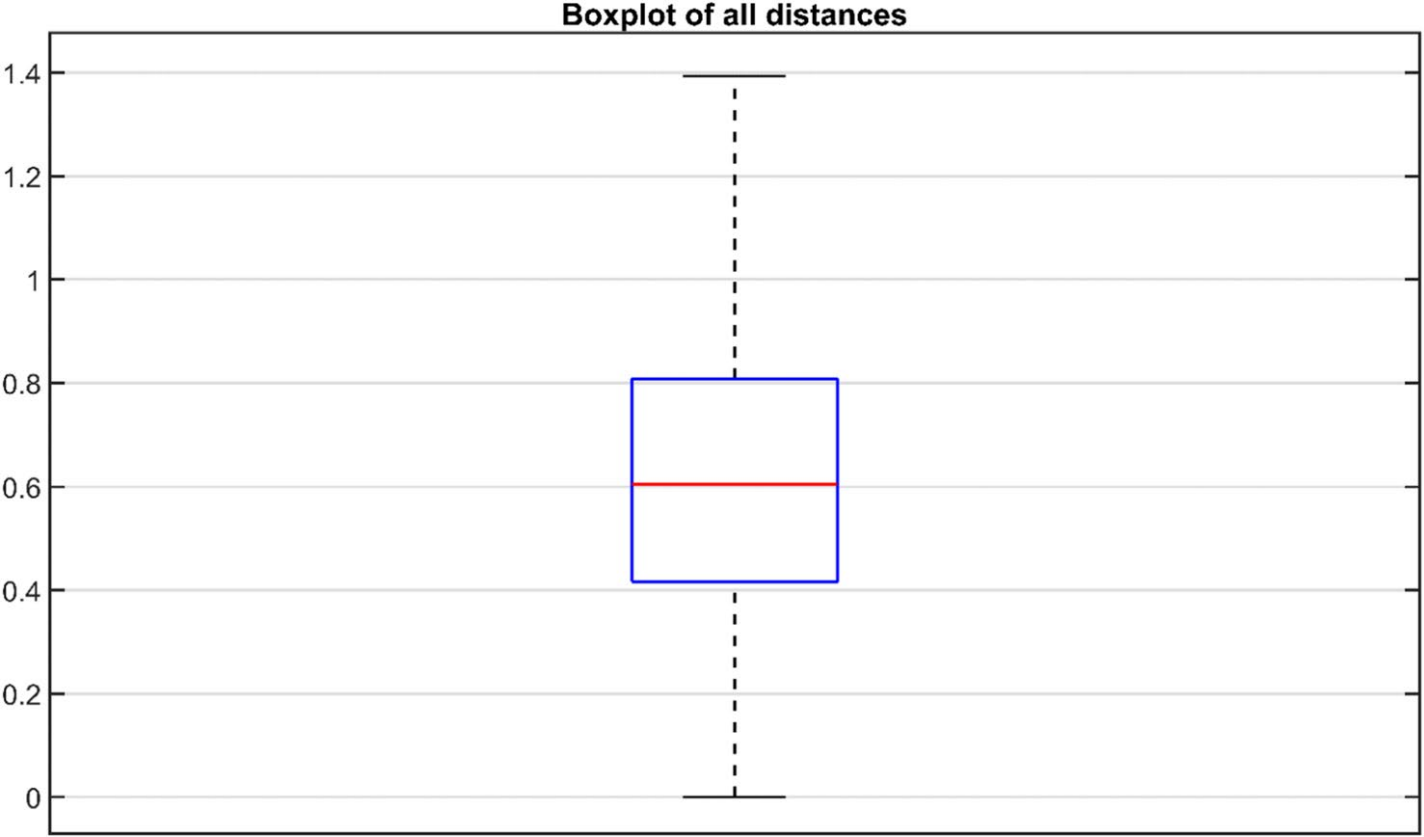
Boxplot of the Euclidean distance of all correspondence points on the left and right tibia

**Fig. 3:**
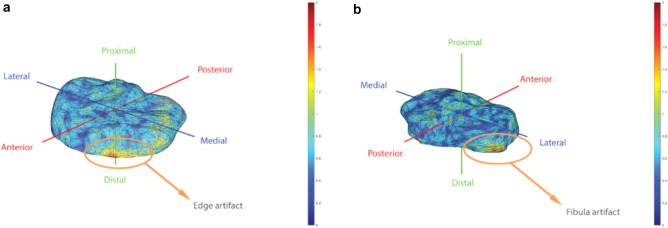
Overview of artefacts: **a** Heatmaps were used to illustrate artifacts at **a** cut-off edges of the tibia and **b** tibiofibular joint

**Fig. 4:**
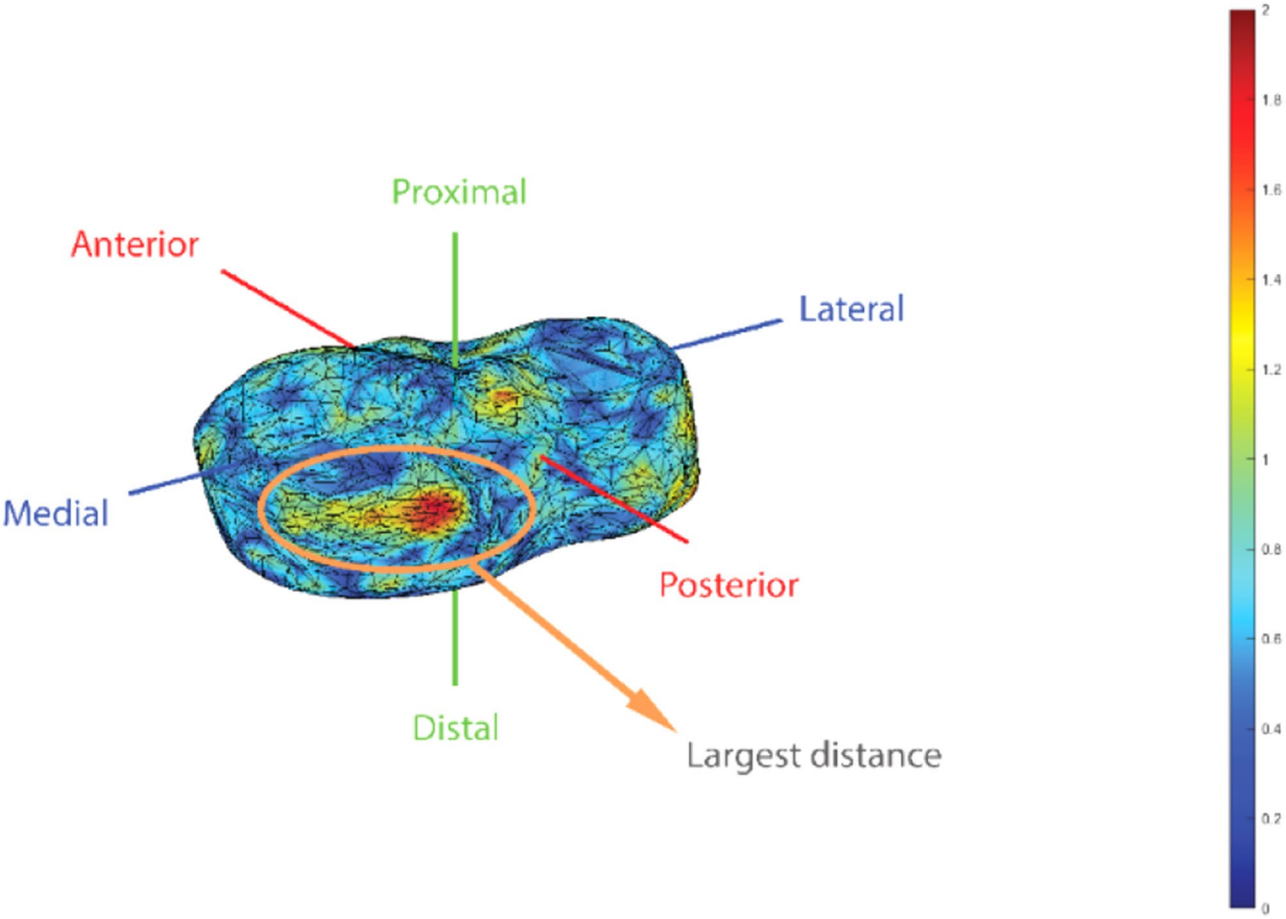
Heatmap investigating the largest distance between correspondence points observed in one subject’s tibial plateau (posterior view)

The mean age of the participants was 24.1 years (range 18–34 years, 71 females, 29 males). The overall mean squared distance between correspondence points on the left and right tibial plateau is 0.6276 ± 0.0343 mm. The differences between all correspondence points were illustrated in a boxplot (Fig. 2). The greatest differences between correspondence points were seen around two specific surfaces of the tibia; where the distal tibia was cut 15 mm below the tibial plateau and around the tibiofibular joint (Fig. 3). The greatest left to right difference, of the subject with the largest mean difference, was 1.6 mm. This difference was found on the medial plateau (Fig. 4).

## Discussion

This study aimed to investigate the equality of the left and right tibial plateau in one hundred healthy living subjects to establish whether mirroring the contralateral tibial plateau can be used to optimize the surgical reduction using 3D virtual planning software for patients with tibial plateau fractures. The overall average distance of correspondence points based on surface matching of the left and right tibial plateau was 0.6276 ± 0.0343 mm.

The distance of 0.6276 mm lies in the range of one voxel size, which was 0.782 × 0.782 × 0.8 mm in this study. To translate this difference into clinical practice; differences in one voxel size are only recognizable in one slice of an axial CT scan of 0.8 mm. Increasing the resolution of the CT scans, could have potentially decreased the distances between the correspondence points. In current literature, the indication for surgical reduction of a tibial plateau fracture varies between a step-off and/or a gap of more than 2–5 mm of the articular surface [4, 19–21]. The average measured distance of 0.6276 mm between correspondence points on the left and right tibia is only a small difference within these clinical margins. Therefore, we are confident that this small difference is not clinically relevant, indicating the contralateral, unfractured, tibial plateau can be used as a template for reduction of the fractured tibial plateau.

Moreover, the knowledge from this study could not only be implemented for 3D virtual planning, but could also be used to address the quality of the postoperative reduction by comparing the postoperative CT scan of the fractured knee and the unfractured contralateral knee. However, for this comparison, it is critical to have access to a CT scan with significant quality to ensure reduction of scattering of the osteosynthesis material. The clinical feasibility of this warrants further research evaluation.

For the participant with the greatest overall left–right difference, there was a localized difference of 1.6 mm on the posterior side of the medial plateau. As Fig. 4 illustrates, the overall distances of the same subject were small, indicating that this is not a superimposing error. This abnormality could be a result of a previous unrecognized posttraumatic injury to the posterior side of the medial meniscus. Despite screening participants for a history of major knee trauma, unrecognized trauma cannot be completely ruled out. In this specific subject, we think this could be a result of twist injury.

A potential limitation of this study is that there were some challenges with the segmentation of the CT scans. The discrimination of bone and soft tissue can be a difficult task in areas with low contrast. For example, around the tibiofibular joint, artificially high distances were be measured due to a poor discrimination of the junction of the tibia and fibula. However, these differences are minimal and do not influence the articular surface of the tibial plateau. Second, cutting the distal tibia 15 mm below the tibia plateau, complicates the determination of correspondence points around this cut off point. This may also have introduced artificially high distances. However, this results only in localized differences, which, due to the high number of total points, only slightly overestimates the average distance of all correspondence points.

Concluding, based on our comparison of one hundred CT scans of the knee in healthy, young individuals without previous damage to the knee, the differences between the left and right tibial plateau are negligible, and therefore, we are confident that the mirrored contralateral, unfractured, tibial plateau can be used as a template for the reduction of a fractured tibial plateau using 3D virtual preoperative planning.
